# Uncovering water conservation patterns in semi-arid regions through hydrological simulation and deep learning

**DOI:** 10.1371/journal.pone.0319540

**Published:** 2025-03-20

**Authors:** Rui Zhang, Qichao Zhao, Mingyue Liu, Shuxuan Miao, Da Xin

**Affiliations:** 1 School of Remote Sensing and Information Engineering, North China Institute of Aerospace Engineering, Langfang, China; 2 Hebei Collaborative Innovation Center for Aerospace Remote Sensing Information Processing and Application, Langfang, China; 3 Hebei Airer Industrial Internet Technology Co., Langfang, China; 4 Langfang Digital Space Technology Co., Langfang, China; Atlantic Technological University, IRELAND

## Abstract

Under the increasing pressure of global climate change, water conservation (WC) in semi-arid regions is experiencing unprecedented levels of stress. WC involves complex, nonlinear interactions among ecosystem components like vegetation, soil structure, and topography, complicating research. This study introduces a novel approach combining InVEST modeling, spatiotemporal transfer of Water Conservation Reserves (WCR), and deep learning to uncover regional WC patterns and driving mechanisms. The InVEST model evaluates Xiong’an New Area’s WC characteristics from 2000 to 2020, showing a 74% average increase in WC depth with an inverted “V” spatial distribution. Spatiotemporal analysis identifies temporal changes, spatial patterns of WCR and land use, and key protection areas, revealing that the WCR in Xiong’an New Area primarily shifts from the lowest WCR areas to lower WCR areas. The potential enhancement areas of WCR are concentrated in the northern region. Deep learning quantifies data complexity, highlighting critical factors like land use, precipitation, and drought influencing WC. This detailed approach enables the development of personalized WC zones and strategies, offering new insights into managing complex spatial and temporal WC data.

## 1. Introduction

Water Conservation (WC) refers to the ability of a region or a specific ecosystem to protect and maintain water resources in its natural state[1]. In semi-arid regions, where precipitation is limited and evaporation rates are high, the natural availability of water resources is severely constrained, making water conservation particularly critical. Safeguarding regional WC is crucial not only for environmental protection but also for economic development[2] and global environmental sustainability[3]. The ecosystems in semi-arid regions, such as sparse vegetation and fragile soils, further complicate water conservation efforts. A scientific understanding of the spatial and temporal distribution of water conservation reserves (WCR) is crucial for regional water resource security[4], ecosystem health[5], and climate regulation[6]. However, due to the combined influence of factors such as climate, topography, soil properties, and land use, the simulation of regional WC is highly dynamic, complex, and uncertain[7]. This poses a significant challenge to the protection of the region’s water resources.

Traditional methods for assessing regional WC primarily rely on manual monitoring of hydrological data, field sampling, and water extraction techniques to evaluate the area’s ability to conserve water resources[8]. However, these methods rely on manual operations, which not only require significant labor but are also vulnerable to factors such as climate change and complex terrain. As a result, the data obtained often have uneven spatiotemporal distribution, leading to deviations in the accuracy of the measurement results. Therefore, traditional methods face greater challenges when facing the large-scale and dynamically changing WC assessment. The development of remote sensing (RS) and geographic information system (GIS) technologies in the field of modeling has led to a more effective assessment of WC[9]. By using RS data and GIS technology, spatial and temporal variations in groundwater resources can be analyzed in more detail, providing a more accurate assessment of WC and overcoming the spatial and temporal constraints and manual labor problems of traditional methods[10]. For decades, the study of WC through “modeling and visualization” has gradually become a new research trend. Current research mainly relies on models based on simulation and analysis of WC, which can be broadly categorized into hydrological models and ecosystem service models. Traditional hydrological models, such as the Soil and Water Assessment Tool (SWAT) model[11] and the Variable Infiltration Capacity (VIC) model[12], focus on runoff simulation and water resource management. They typically emphasize physical processes like dynamic changes in water resources, pollutant diffusion, and soil moisture distribution, but lack quantitative and comprehensive assessments of ecosystem service values. The Integrated Valuation of Ecosystem Services and Trade-offs Model (InVEST)[13] focuses on the quantification and visualization of ecosystem services, enabling the quantitative assessment and multidimensional analysis of ecosystem services. It is especially useful in fields such as environmental protection, sustainable development, and ecological restoration, as it integrates ecological, economic, and social factors to provide more comprehensive decision support[14]. However, most studies[15–17] using the InVEST model to assess regional WC primarily focus on evaluating the overall WC capacity of the region, with limited analysis of the temporal changes in WC within the same region. Our goal is to fill this gap by classifying WCR into different levels and analyzing trends in these changes. This approach will guide land use planning, assess the priority and development of WCR.

Another challenge lies in unearthing the driving mechanisms of WC, and while meteorological[18]and soil factors[19,20]have been recognized as important predictors of WC, few observations have been made of the cross-correlation of each factor with WC. This may be due to the nonlinear relationships[21,22]between many parameters and WC, which complicates the determination of simple linear relationships between parameters and WC. In analyzing the drivers, most scholars usually use traditional statistical methods[23–25]. Jia, et al.[26]used principal component analysis to identify precipitation, evapotranspiration, and forest and grasses as the main drivers affecting water yield. Alireza Daneshi and Yanqing Lang et al.[27,28] Use of scenario analysis to assess the impact of climate and land use on regional WC. While these method help to understand the main patterns of change in the data, it does not allow for a specific degree of influence of each characteristic on the target variable. In the face of large datasets, it is difficult for these methods to account for nonlinear relationships. In recent decades, machine learning has gained popularity in water resources management[29], as it can effectively handle nonlinear processes[30,31]. Xie, et al.[32]used a tree-modeling importance analysis to identify weather as the main driver influencing changes in WC, which not only accounted for nonlinear effects, but also provided a specific degree of influence of each characteristic, but it still did not account for the factors’ interactions. In addition, most of the studies[16,33,34]only discuss the influence mechanism of the main drivers and lack detailed analysis of the specific influence mechanism of individual factors on WC capacity. In contrast, deep learning is uniquely positioned to analyze complex ecosystems through its multilayer neural network structure[35]. Despite significant progress in other areas, deep learning is still less frequently applied to assess and analyze water cycle drivers[36,37]. In the field of WC assessment, decision makers need a clear understanding of model-driven mechanisms in key decision areas such as ecosystem stability and WC management in order to develop reliable management strategies. However, there is a lack of physical explanation of the deep learning model assessment process[38]. Research has increasingly shifted towards explainable artificial intelligence (XAI) technologies. XAI helps users understand the driving factors behind model predictions by revealing the importance of features and their relationships[39]. Techniques like Shapley additive explanations (SHAP) and Local Interpretable Model-agnostic Explanations (LIME) have been applied to interpret black-box models. LIME focuses on providing local explanations[40], Grad-CAM is useful for image feature interpretation[41], and Individual Conditional Expectation (ICE) offers limited readability under multidimensional features and cannot provide global feature importance. In contrast, SHAP does not have these limitations. Derived from cooperative game theory, SHAP is used to determine each participant’s contribution to a collaborative task. It can provide local explanations for individual samples and global explanations across the entire dataset. Therefore, we aim to fill this gap by modeling the solution of the regional WC drivers and introducing XAI for interpretation. K-fold cross-validation and Monte Carlo were utilized to verify the accuracy and stability of the model.

This study focuses on the Xiong’an New Area in Hebei Province, China, exploring the spatiotemporal patterns of water conservation (WC) in a semi-arid region and its driving factors. Unlike traditional analysis methods, this research incorporates the InVEST model to include the perspective of critical zone transitions, offering a comprehensive analysis of the spatiotemporal distribution of regional WC and water conservation reserves (WCR). Additionally, by integrating deep learning and explainable artificial intelligence (XAI) techniques, the study reveals the underlying driving mechanisms. The introduction of deep neural networks (DNN) and XAI methods, particularly the SHAP model, not only enhances the accuracy of hydrological analysis but also effectively addresses nonlinear and uncertain factors, overcoming the limitations of traditional hydrological simulations in handling complex data. This approach improves the transparency and interpretability of the model, providing new insights into WC and assisting policymakers in formulating more precise water resource management strategies to promote regional sustainable development.

## 2. Materials and methods

### 2.1. Study area

**Fig 1:**
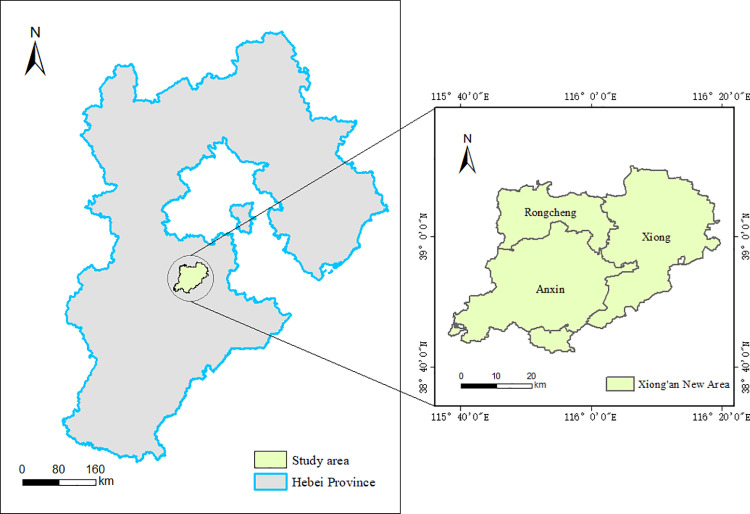
Overview of the study area. (Data from OpenStreetMap and its contributors).

In this study, the objective is to assess the water conservation (WC) of Xiong’an New Area in Hebei Province and investigate the driving factors influencing it (Fig 1). Located near the heart of Hebei Province (38°43’N to 39°10’N, 115°38’E to 116°20’E), Xiong’an New Area sits on the alluvial fan of the Daqing River system[42]. It encompasses Xiong County, Rongcheng County, Anxin County, and parts of the neighboring regions[43], with elevations ranging from 7 to 19 meters. The area features a gently sloping plain with an open topography, characterized by several ancient river channels. As a semi-arid region, it also hosts the largest freshwater wetland in northern China[44], which plays a crucial role in local hydrology and ecology.

### 2.2. Data sources and processing methods

Table 1 outlines the data sources used for calculating, validating, and assessing the contribution to WC. The selected data primarily includes soil and meteorological information, with land use data spanning seven periods: 2000, 2005, 2008, 2010, 2015, 2018, and 2020. Root restricting layer depth data is sourced from the ISRIC SoilGrids 250m (2017 version) depth-to-bedrock (R layer) dataset, while plant available water capacity is consolidated into a single layer using the global SoilGrids 2017 AWC data and integrated into the Hengl equation[45]. Additional data processing follows the InVEST 3.13.0 manual. The preprocessed data was then input into the InVEST model, and the final model results were mapped.

**Table 1:** Data sources.

Data Type	Description	Data Source
Land use data	Land use/cover change	Resource and Environmental Science Data Platform (http://www.resdc.cn/)
Climate data	Precipitation, potential evapotranspiration, dryness, mean annual temperature	National Tibetan Plateau Data Center (https://data.tpdc.ac.cn)
Soil data	Root restricting layer depth Plant available water content(PAWC) Root depth	Harmonized World Soil Database (https://www.fao.org/soils-portal/en/)
Topographic data	DEM data	Geospatial Data Cloud (https://www.gscloud.cn/)
Vegetation data	Normalized Difference Vegetation Index(NDVI) Fractional Vegetation Cover(FVC)	National Science and Technology Infrastructure (http://www.nesdc.org.cn) National Tibetan Plateau Data Center(https://data.tpdc.ac.cn)

### 2.3. Dataset

In the data preprocessing, points with a median value of 0 for water retention were removed, a sample buffer was created, factor values for each year as well as water retention were extracted into the sample points, and outliers were averaged to create an initial database of natural factors, for a total of 3,438 spatial point datasets. The dataset contains seven characteristic factors such as P,AI, TEM, PET, NDVI, FVC and LUCC, aiming to quantify their effects on water stocking (Table 2).

**Table 2:** List of indicator types and abbreviations.

Type	Indicator	Abbreviation	Unit
Climate	Average precipitation	P	mm
	Annual arid index	AI	–
	Average temperature	TEM	°C
	Annual potential evapotranspiration	PET	mm
Vegetation	Normalized Difference Vegetation Index(NDVI)	NDVI	–
	Fractional Vegetation Cover	FVC	–
Land Use	Land use/cover change	LUCC	km²

### 2.4. Methods

**Fig 2:**
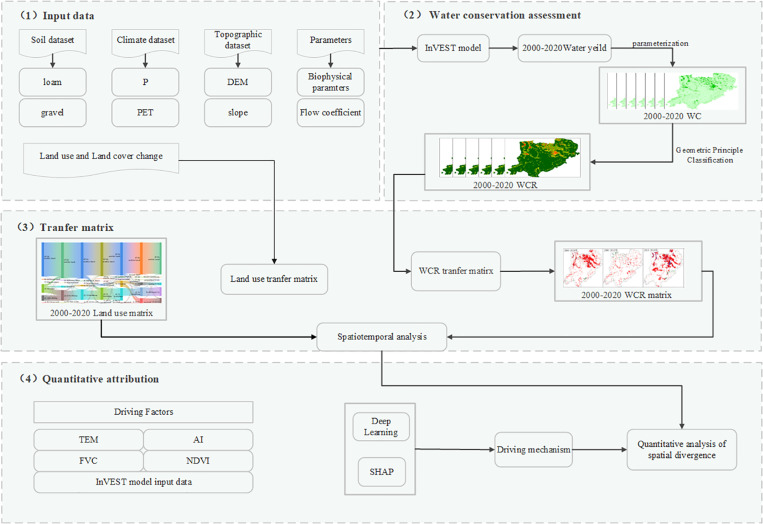
Technical lines of study.

WC is adjusted using a hydrological model to obtain accurate values. The transfer matrix method and partitioning technique are then employed to assess the spatiotemporal dynamics of WC and WCR. Additionally, WC predictions are made by constructing a sample dataset for input into a deep learning model. Finally, the SHAP model is applied to explain the relationships between variables and predicted values. Fig 2 illustrates the technical approach of the study. The following sections will detail the specific modeling methods used.

#### 2.4.1. InVEST model.

The InVEST model is used for ecosystem service analysis and optimization[46]. The water yield module[47]of the InVEST model can be used to assess WC. After the model obtains the water yield, it is corrected using parameters such as the terrain index, and ultimately the WC is obtained. The Water Yield module estimates how water resources are distributed across various landscape areas, using factors like the Budyko curve and average annual precipitation. The module employs a water balance approach, considering soil properties, precipitation patterns, surface evapotranspiration, land use, and other variables. The tool employs a new method that considers the spatial variability of soil characteristics. It assesses the maximum soil water content.

The formula for calculating the annual water production for each pixel in the model:


$$\text{Y}\left(\text{x}\right)=\left(1-\frac{\text{AET}\left(\text{x}\right)}{\text{P}\left(\text{x}\right)}\right)\text{*P}\left(\text{x}\right)$$ (1)

In the equation, x represents the pixel. AET(x) denotes the annual actual evapotranspiration for x. P(x) indicates the annual precipitation for x.

The corrected formula for the WC quantity is:


$$\text{WCA}=\text{Min}\left(249∕\text{V},1\right)\text{*Min}\left(1,0.9\text{*}\frac{\text{TI}}{3}\right)\text{*Min}\left(1,\frac{\text{K}}{300}\right)\text{*Y}$$ (2)

$$\text{TI}=\log\left(D∕S\times P\right)$$ (3)

$$K=1.148\times 10^{\left(-0.6+1.26*0.01*c2-6.4*0.001*c1\right)}$$ (4)

WC represents the water recharge of the study area in millimeters (mm); TI represents the topographic index; V represents the flow coefficient; K represents the saturated hydraulic conductivity of the soil in centimeters per day (cm/day), calculated using the Cosby soil transfer[48]equation Y represents the water yield calculated by the model in millimeters (mm); D represents the number of grids in the catchment; S represents the depth of the soil layer in millimeters (mm); P represents the percent slope; c1 and c2 are the clay and sand content of the soil (%), respectively.

#### 2.4.2. Classification.

According to the method of grading the importance of ecosystem services in the National Ecological protection Red Line - Technical Guidelines for the Delineation of Ecological Functions Red Line[49]. The relative contribution of different areas to water resources is assessed using the water yield module of the InVEST model. In order to better analyze the spatial and temporal evolution characteristics of WC in Xiong’an New Area, the class classification was carried out according to the principle of geometric intervals. Geometric intervals create class intervals through geometric sequences, and use geometric ratios to divide the data into a number of classes, and the formula for geometric ratios is:


$$r={\left(\frac{Max\left(x\right)}{Min\left(x\right)}\right)}^{\frac{1}{k}}$$ (5)

r represents the geometric ratio. Max(x) and Min(x) denote the maximum and minimum values of the divided ranks, respectively k stands for the number of divided ranks..

The formula for each interval is:


$$Interval_{i}=\left(Min\left(x\right)\cdot r^{\text{i}-1},Max\left(x\right)\cdot r^{\text{i}}\right)$$ (6)

where *i* ranges from 1 to k, the goal is to minimize the sum of the squares of the number of elements in each class.

#### 2.4.3. Driver assessment model.

**Fig 3:**
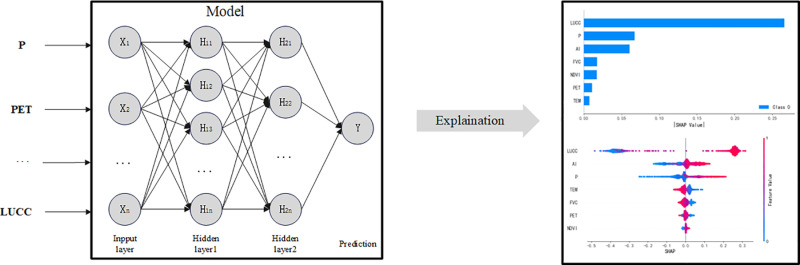
Model architecture.

This study uses a deep neural network (DNN) to predict streamflow conservation volume, aiming to learn and represent complex patterns within the data(Fig. 3)[50]. The constructed DNN model utilizes the TensorFlow and Keras frameworks and consists of an input layer with 7 nodes and three fully connected layers (the first layer with 10 neurons, the second layer with 5 neurons). Each layer uses the ReLU activation function, and dropout layers are introduced after each fully connected layer to reduce the risk of overfitting. The output layer is a single-node dense layer responsible for predicting the streamflow. The model is compiled using the Adam optimizer and mean squared error (MSE) as the loss function, and it undergoes 1,000 epochs of training to effectively learn the complex patterns in the data. Additionally, 5-fold cross-validation is used to evaluate the model’s performance, calculating R² values, mean squared error (MSE), and mean absolute error (MAE) for both the training and test sets. Uncertainty analysis is conducted using the Monte Carlo dropout method, predicting the standard deviation of the results as an uncertainty metric, providing a comprehensive understanding of the model’s predictions.

#### 2.4.4. Explainable artificial intelligence (XAI).

In machine learning, black-box models refer to those whose internal mechanisms are difficult to understand or interpret, such as deep learning models and ensemble methods. These models often achieve high predictive accuracy, but due to their complexity, it is challenging to understand how features influence the final prediction. The goal of Explainable Artificial Intelligence (XAI) is to enhance the transparency of machine learning models, making their decision-making processes more understandable[51]. XAI focuses on uncovering the internal mechanisms of models to help users understand how specific decisions are made. SHAP (SHapley Additive exPlanations) is a game-theory-based explanation method that provides consistent and reasonable feature importance explanations for any machine learning model[52]. SHAP combines feature importance and local interpretability, assigning each feature a contribution value that reflects its impact on the model’s output. The core idea of SHAP is based on Shapley values, which originate from cooperative game theory. Shapley values are used to fairly allocate the contributions of participants in a cooperative setting, ensuring that each participant’s reward is proportional to their contribution. SHAP calculates the marginal contribution of each feature across different combinations of features, assigning an importance value to each feature[53].

The calculation of the SHAP value can be expressed by the following formula:


$$\varphi_{i}\left(f\right)=\underset{S\subseteq N\left\{i\right\}}{\sum }\frac{\left|S\right|!\cdot \left(\left|N\right|-\left|S\right|-1\right)!}{\left|N\right|!}\left[f\left(S\cup \left\{\text{i}\right\}\right)-f\left(S\right)\right]$$ (7)

$\phi_{i}\left(f\right)$ is the SHAP value for feature i; *N* is the set of all features; S is any subset of features that does not include i. $f\left(S\right)$ is the model's prediction using the feature subset S.

## 3. Results and discussions

### 3.1. Changes in LUCC

**Fig 4:**
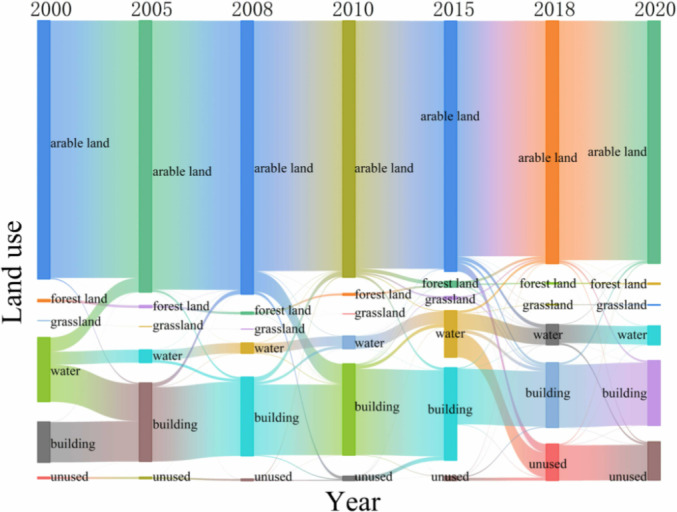
Changes of land types.

Table 3 and Fig 4 present the land use statistics for Xiong’an New Area from 2000 to 2020. They indicate that cropland is the predominant land type in the area. The management and use of this land type significantly affect WC. Effective management of arable land not only preserves soil’s hydrological functions but also supports a healthy water cycle. By boosting vegetation cover and enhancing soil structure, it helps maintain and improve the area’s WC. Its area shows a trend of increasing and then decreasing, reaching a maximum in 2008, with an area of 1,329.05km², accounting for 74% of the area, but then gradually decreasing, mainly transforming into construction land and forest land; grassland and forest land cover can filter and purify pollutants in precipitation, reduce the pollution of the water body by agricultural and urban discharges, and maintain the health of water quality and the ecosystem. The area of woodland and grassland reached its maximum in 2015, mainly due to nature reserves and ecological compensation policies [54], as well as the implementation of ecological measures such as afforestation[55]. However, the area of water bodies decreased by 218.98 km², primarily being converted into building and unused land. Meanwhile, construction land increased by 119.59 km² and unused land rose by 177.72 km². These expansions mainly resulted from the conversion of water bodies and arable land.

**Table 3:** Changes of land types and area in Xiong’an New Area from 2000 to 2020/km².

Year Land use type	2000	2005	2008	2010	2015	2018	2020
Arable land	1255.06	1318.85	1329.05	1246.25	1217.02	1180.03	1178.56
Forest land	12.00	11.94	11.57	9.62	25.36	7.05	6.83
Grassland	0.51	0.52	0.52	0.42	15.88	3.76	3.76
Water body	312.34	243.43	230.34	214.35	226.9	100.52	93.36
Building	196.32	201.65	204.67	292.46	277.41	315.97	315.91
Unused	9.29	9.13	9.32	22.42	22.91	178.18	187.01

### 3.2. Analysis the results of WC

#### 3.2.1. InVEST model accuracy validation.

**Fig 5:**
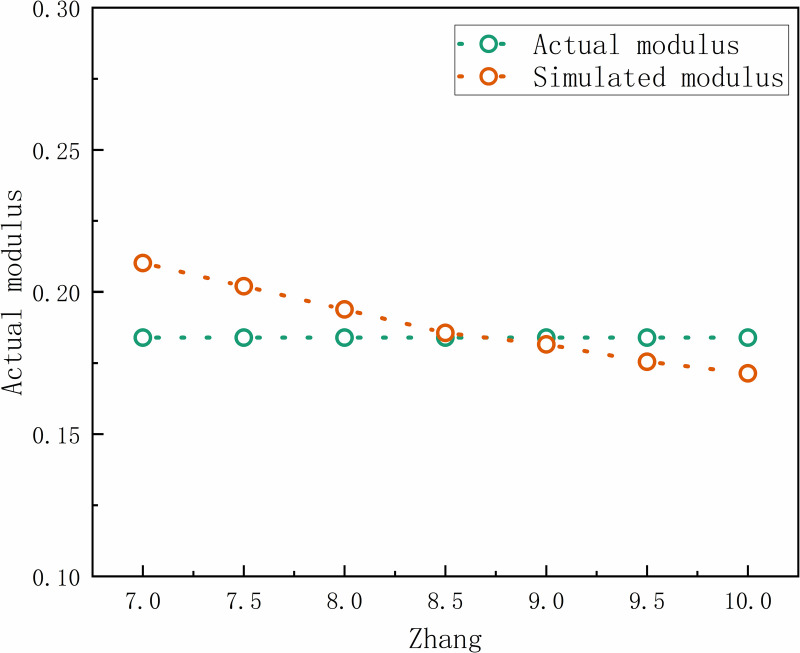
Model verification result.

In the case of determining the input data for the model, the seasonal factor tensor coefficient is one of the important parameters affecting the accuracy of the model, and its value usually varies between 1 and 30. Based on the water production modulus of Xiong’an New Area in the Hebei Province Water Resources Bulletin (2020) as the standard data, we carried out a detailed test and correction of the model’s calculation results to ensure its fit with the actual monitoring data. Fig 5 illustrates the model correction process data. The study results show that setting Zhang to 8.5 yields the following outcomes. The water yield calculated by the model aligns best with the actual monitoring data, achieving a correlation coefficient (R²) of 0.991. This indicates that there is a significant linear correlation between the Zhang coefficient and the modal number of water yield, which verifies the importance and accuracy of the Zhang coefficient in the model.

#### 3.2.2. Features of the spatiotemporal distribution of WC.

**Fig 6:**
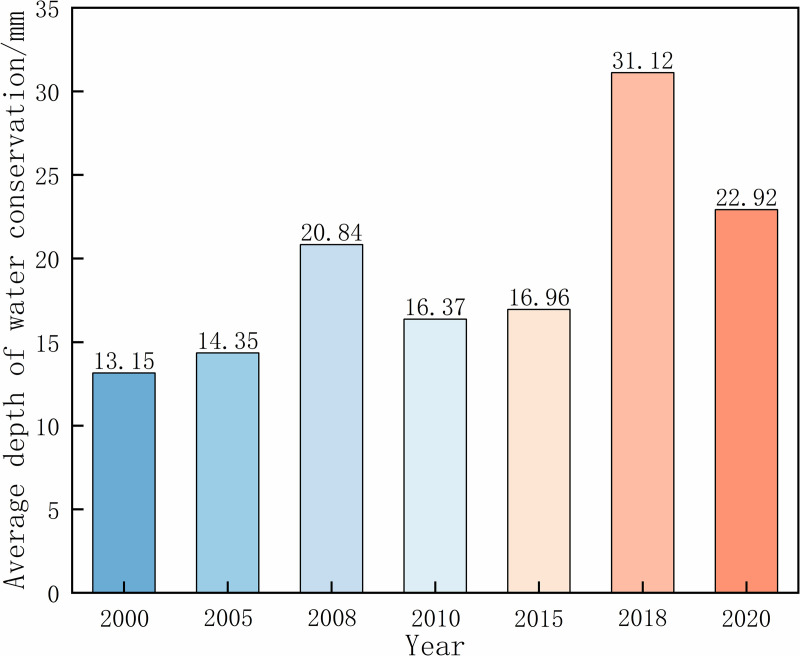
Average depth of WC in Xiong’an New Area from 2000 to 2020.

Based on the accuracy of the model calibration, we further analyzed the spatiotemporal pattern of WC in Xiong’an New Area. From a temporal perspective (Fig 6), WC in Xiong’an New Area showed a fluctuating upward trend, with the overall growth rate slowing down and then accelerating, with a total growth rate of 74%. Specifically, WC reached two peaks in 2008 and 2018, at 20.84 mm and 31.12 mm, respectively. This indicates that the average depth growth rate of WC was the highest during the construction phase of the new area (2015-2018), at 83%, indicates the effectiveness of policy implementation and the increase in public environmental awareness. Overall, the regional WC is gradually improving, and the growth trend indicates the positive impact of ecological construction and management measures on WC.

**Fig 7:**
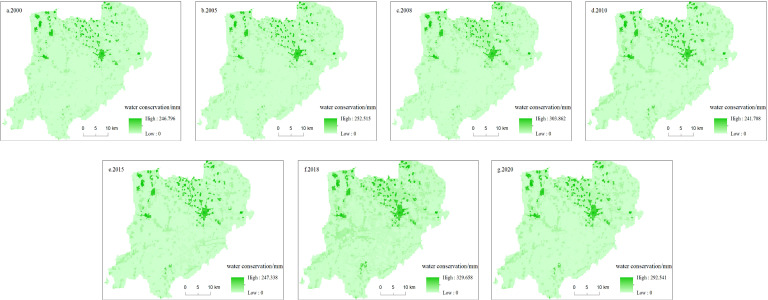
Spatial distribution of WC from 2000 to 2020.

In terms of spatial distribution (Fig 7), the WC in Xiong’an New Area forms an inverted “V” shape. A high-value area develops from the southwest to the northeast. This distribution pattern indicates that, with the continuous promotion of ecological restoration and ecological civilization construction, the range of high WC areas gradually expanded, mainly concentrated in the areas with high values of vegetation index in Xiong and Rongcheng counties[56]. This change reflects the remarkable effectiveness of vegetation restoration and ecological construction in the region, and further verifies the important role of ecological restoration measures in enhancing WC capacity.

### 3.3. Analysis of WCR

**Fig 8:**
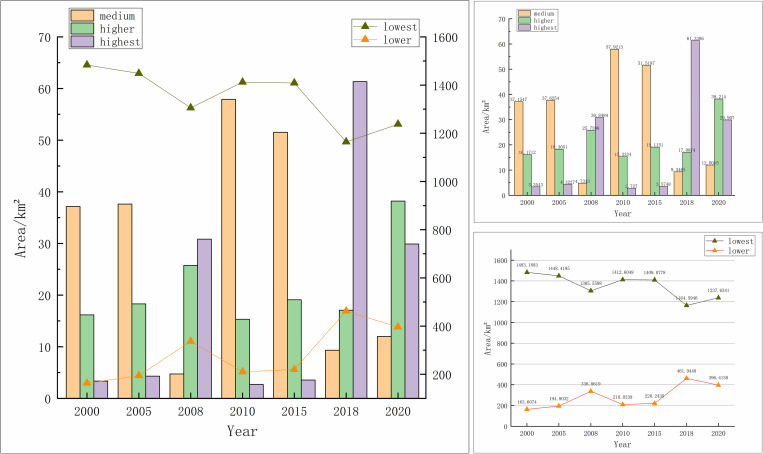
Overall (a) and partial plots (b,c) of area occupied by WCR from 2000 to 2020.

Classification of the amount of WC can get the WCR. In this study, the average value of the geometric interval division value was used as the division interval to ensure the continuity of the division. The range of specific zones is as follows: lowest WCR (0-21.40 mm), lower WCR (21.40 mm-74.76 mm), medium WCR (74.76 mm-182.69 mm), higher WCR (182.69 mm-212.62 mm), and highest WCR (212.62 mm-329.66 mm). Table 4 and Fig 8 show the statistical results of the area of WCR in Xiong’an New Area. The data show that the area of lowest WCR continues to decrease, totaling 245.56 km², and this result indicates that the lowest WCR is changing at a larger magnitude, and there is a greater potential for advancing the WCR flow. Especially in 2015-2018, lowest WCR area decreased at the fastest rate. In addition, lower WCR, higher WCR and highest WCR areas showed an overall increasing trend, increasing by 232.81 km², 22.04 km² and 26.56 km², respectively. This shows that the WCR enhancement measures in these areas are effective and can help the region to improve the WC. medium WCR area is wavering, so the status quo can be maintained first, and we can grasp the pattern and prescribe the right medicine. To summarize, the lowest WCR and lower WCR are the most suitable for rectification.

**Table 4:** Grade change transfer matrix in important areas of WC/km².

Year	WCR	Lowest	Lower	Medium	Higher	Highest
2000-2005	Lowest	1472.03	7.87	2.16	1.09	0.04
	Lower	17.67	145.52	0.32	0.07	0.03
	Medium	0.09	0	33.34	3.72	0
	Higher	0.03	0.01	1.81	13.02	1.31
	Highest	0	0	0	0.41	2.94
2005-2008	Lowest	1299.64	187.05	0.17	0.94	1.04
	Lower	4.71	147.93	0.28	0.1	0.3
	Medium	0.08	1.02	4.25	24.53	7.72
	Higher	0.01	0.48	0.02	0.17	17.62
	Highest	0	0.14	0.01	0.02	4.16
2008-2010	Lowest	1224.87	71.13	1.66	0.02	0.01
	Lower	179.61	137.83	13.57	3.82	0.51
	Medium	0.39	0.25	4.02	0.01	0.01
	Higher	2.25	0.07	23.31	0.1	0.01
	Highest	1.43	0.49	15.29	11.38	2.19
2010-2015	Lowest	1379.59	26.35	2.01	0.58	0.13
	Lower	16.89	192.6	0.08	0.23	0.02
	Medium	3.97	0.06	48.78	5.02	0.04
	Higher	0.95	0.07	0.17	13.21	0.92
	Highest	0.2	0.02	0.03	0.01	2.47
2015-2018	Lowest	1133.41	261.48	3.1	2.85	6.93
	Lower	19.8	195.68	3.83	0.08	0.69
	Medium	1.73	1.4	2.26	13.94	32.15
	Higher	0.49	0.42	0	0.18	18.02
	highest	0.11	0.09	0	0.02	3.35
2018-2020	Lowest	1154.26	8.77	0.08	0.05	0.04
	Lower	78.69	380.44	0.4	1.29	0.85
	Medium	2.1	4.34	2.89	0.01	0.01
	Higher	1.03	0.57	7.81	7.49	0.17
	Highest	0.33	1.99	0.81	29.36	28.83

**Fig 9:**
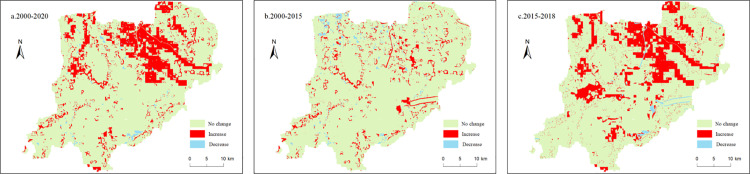
Grade change of important WC areas in Xiong’an New Area.

Table 4 and Fig 9 show the statistical results of the change of WCR in Xiong’an New Area, and the data show that most of Xiong’an New Area belongs to the lowest WCR, but its area has been decreasing. At the same time, the area of lower, medium, higher and highest WCR has been increasing continuously, and the area of the lowest transformed to lower WCR is as high as 261.48 km² in the period of 2015-2018. Especially in the period of 2018-2020, the area of lower and highest WCR increased significantly, indicating that the WC function of Xiong’an New Area is improving. In the preconstruction period of Xiong’an New Area (2000-2015), the WCR area was scattered and not well-defined. However, during the construction period (2015-2018), there was a significant increase in the WCR area, accompanied by a rapid improvement in water source containment capacity, especially in the central and northern regions. This notable improvement highlights the success of various ecological protection measures implemented in Xiong’an New Area. These measures include the delineation of the ecological red line, comprehensive water pollution treatment, and other initiatives aimed at environmental conservation[57].

### 3.4. Analysis of the driving mechanisms of the WC

#### 3.4.1. Model performance evaluation.

**Fig 10:**
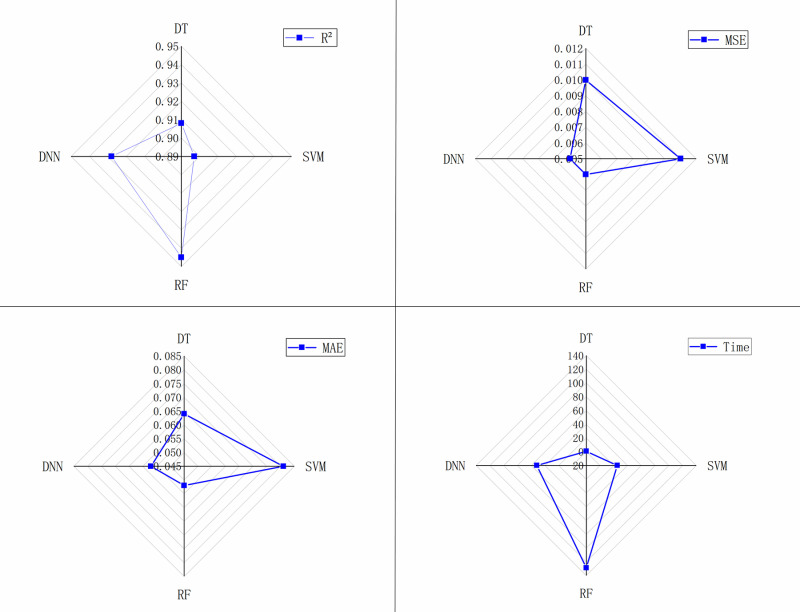
Model evaluation results.

To comprehensively assess the performance of various models in predicting water conservation (WC), a detailed evaluation was conducted using multiple metrics[29,58]. The performance metrics include the coefficient of determination (R²), root mean square error (RMSE), mean absolute error (MAE), and model runtime (Time). R², RMSE, and MAE are employed to evaluate the models’ goodness of fit, while runtime reflects the computational cost of each model. All experiments were performed on the same hardware and dataset to ensure consistency. As shown in Fig 10, the evaluation results indicate that the Random Forest (RF) and Deep Neural Network (DNN) models excel in terms of goodness of fit. Meanwhile, the Decision Tree (DT) and Support Vector Machine (SVM) demonstrate the lowest computational cost for both training and prediction, highlighting their efficiency in terms of computational complexity.

In the study of WC driving factors, there is typically a nonlinear relationship between WC and its driving factors. While DT and SVM can handle high-dimensional and complex data, they are prone to overfitting and struggle to effectively capture such nonlinear relationships. Although RF and DNN are better equipped to handle the multidimensionality and nonlinear characteristics of data, RF exhibits significantly higher computational complexity compared to DNN. As the size of the dataset increases, RF’s resource consumption escalates, making it less scalable. While DNN models do involve relatively higher computational costs, these costs remain within an acceptable range and are offset by their superior fitting capability and excellent generalization performance. Therefore, based on a comprehensive evaluation of model computational complexity, training time, accuracy, and data characteristics, DNN is selected as the core method for fitting multifactor relationships in this study.

To improve the stability and generalization of the model, we use cross-validation and Monte Carlo simulation. In cross-validation, the dataset is divided by time, the model is trained and sexually validated on each subset, and the results are averaged. Monte Carlo simulation quantifies the stability of the predictions by random sampling and calculating the standard deviation of the model output. To thoroughly evaluate the driving mechanisms of WC, it is essential to consider both natural environmental factors and socio-economic factors together. From the perspective of InVEST model input, P, PET and LUCC data were selected as driving factors, while the model only considered natural factors and ignored the interference of anthropogenic factors, so four factors, namely, NDVI, FVC, AI and average annual TEM, were supplemented. According to Table 5, the deep learning models constructed by selecting the above seven factors have an average mean absolute error (MAE) less than 0.09, an average coefficient of determination (R²) greater than 0.90 for both the training and test sets, and the average model uncertainty, as indicated by the standard deviation, is below 0.11, indicating that the deep learning model can explain the driving force of each factor on WC.

**Table 5:** Model training accuracy.

Indicator Index Year	Average Training R^2^	Average Testing R^2^	Average MAE	Average Uncertainty Standard Deviation
2000	0.9124	0.9086	0.0767	0.1006
2005	0.9320	0.9124	0.0818	0.1092
2008	0.9138	0.9074	0.0674	0.0901
2010	0.9123	0.9049	0.0765	0.1016
2015	0.9501	0.9392	0.0656	0.1014
2018	0.9140	0.9064	0.0770	0.0907
2020	0.9297	0.9202	0.0787	0.1076
2000-2020	0.9289	0.9131	0.0749	0.0984

#### 3.4.2. Analysis of drivers of WC.

**Fig 11:**
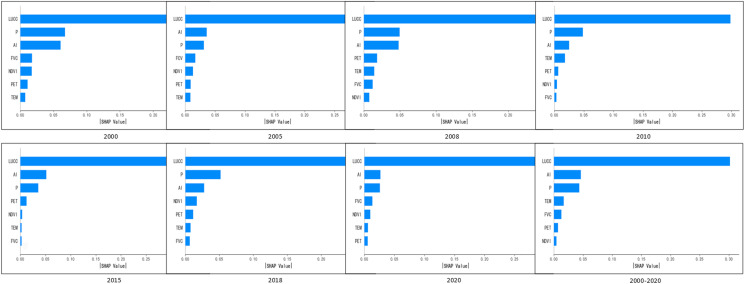
Evaluate the significance of features across different years.

To further explore the influence of various factors on WC, we analyze the SHAP values for each driving factor in the neural network model. Fig 11 shows the mean value of the absolute value of SHAP of each driving factor in the neural network model, the higher the corresponding value of the factor, the stronger the driving force of the feature on WC. The results show that the LUCC has the greatest influence, and its importance is always in the first place, followed by P and AI, and the influence of the four factors, NDVI, FVC, annual average TEM and PET, fluctuates between the 4th and 7th places, and the specific ranking changes with time.

#### 3.4.3. Analysis of the response of the WC nutrient quantity to different factors.

**Fig 12:**
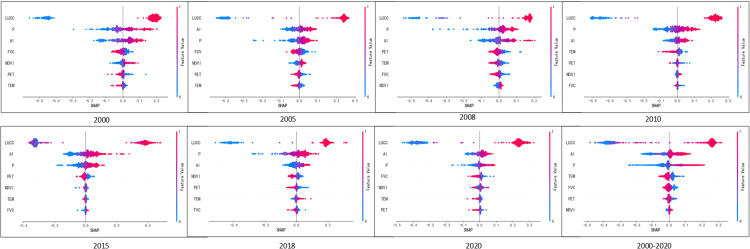
Plot of SHAP summary plot.

SHAP can provide not only the importance of global features, but also the physical interpretation of individual features. This study selected 3,438 experimental sites to construct a summary graph of driving factors, examining how changes in individual factor values relate to WC. As shown in Fig 12, each row represents a feature, and each point represents a sample. The value range of each factor is displayed using normalized results. The LUCC data are arranged as follows: arable land, forest land, grassland, watershed, building land (including urban land, rural residential land, industrial and commercial construction land, and bare land), and unused land (including wetland, marshland, and tundra).

As analyzed in Fig 12, land use types exhibit a positive correlation with water conservation (WC). Low WC values are concentrated in arable land types. Arable land exposes the soil surface, which is prone to being washed away by rainwater, leading to soil erosion and reduced WC capacity. On the other hand, the root systems of forested and grassland areas help stabilize the soil and enhance the permeability of the system, making it easier for water to infiltrate into the groundwater layer. Lakes, with their abundant vegetation such as watercress and reeds, can regulate water temperature by blocking sunlight, which prevents the water from becoming too hot. These plants also help adsorb suspended materials and other substances, purifying the water and maintaining the lake’s ecosystem health. Built-up areas often seal the land and increase surface runoff, but the surrounding green belts usually present in residential areas can improve the region’s WC. Wetlands positively impact WC by absorbing and storing precipitation, slowing down rainfall drainage, and contributing to ecological balance and biodiversity protection[23].

AI, or aridity index, is defined as the ratio of evapotranspiration to precipitation and is positively correlated with WC. In regions where precipitation is generally low and evapotranspiration is high, WC can be improved by capturing rainwater during the dry season to support vegetation growth and groundwater recharge. However, in extreme dry conditions, the rate of soil water evapotranspiration may significantly exceed the rate of precipitation recharge, leading to a decrease in WC. AI reflects the degree of dryness in a region, with higher AI values indicating greater aridity and more pronounced challenges for water conservation. To address these challenges, effective management of soil and vegetation is essential. Strategies such as enhancing soil moisture retention, optimizing land use practices, and increasing vegetation cover can help mitigate the negative impacts of high evapotranspiration. By implementing these measures, regions can better sustain or even improve their water conservation efforts despite high levels of dryness.

Large amounts of P can increase soil moisture recharge, slow the rate of soil moisture depletion, promote the replenishment of underground water and surface water, and provide sufficient water sources for vegetation growth. This occurs because ample P allows for greater infiltration of water into the soil, which replenishes both soil moisture and groundwater reserves, and reduces runoff, thereby enhancing water availability for vegetation. However, higher P does not necessarily translate to higher WC in absolute terms. Other factors, such as LUCC, topography, and FVC, play crucial roles. For example, in regions with undulating topography, water may run off quickly rather than infiltrating into the soil. Poor soil permeability can hinder the infiltration process, leading to surface runoff rather than water retention. Insufficient FVC can reduce the amount of water that is captured and retained by the soil[59]. Therefore, even in areas with high P, WC may remain low if these factors are not conducive to water retention and conservation.

The results revealed that factors such as FVC, mean annual TEM, NDVI, and PET had a relatively minor effect on the model outcomes. Dense vegetation is vital for WC because it captures rainwater, reduces runoff, prolongs the water’s residence time on the surface, enhances infiltration into the soil, boosts soil water reserves, and replenishes groundwater and lakes, thereby significantly improving regional WC. Variations in air TEM can influence regional precipitation patterns and soil moisture evapotranspiration rates; for instance, higher TEM can increase PET, potentially reducing soil moisture and hindering WC. The NDVI serves as an important indicator of vegetation health, with higher values generally reflecting robust vegetation growth that positively contributes to WC by improving water retention and reducing runoff. Conversely, high PET rates suggest increased surface water loss, greater soil dryness, and diminished vegetation transpiration, which can adversely affect WC by decreasing the amount of water retained in the soil. Thus, understanding these factors and their impact mechanisms is crucial for developing effective water conservation strategies.

## 4. Conclusions

This study reveals the primary water conservation (WC) patterns in semi-arid areas from three perspectives: natural resources, spatiotemporal variation, and spatial zoning. First, the InVEST model was used to estimate WC and conduct spatiotemporal analysis, while a transition matrix was employed to identify the main WC patterns in the region. Secondly, a combined model of Deep Neural Networks (DNN) and SHAP was used for a comprehensive analysis of the driving factors. The main research findings are as follows:

Spatiotemporal Evolution of WC and WCR: Between 2000 and 2020, the total WC in Xiong’an New Area averaged 3.432 million m³, showing an overall increasing trend. This indicates that WC efforts in the region have been gradually strengthening. Spatially, WC exhibited an inverted “V” pattern from southwest to northeast, with high-value areas concentrated in the central regions of Xiong County and Rongcheng County. This distribution suggests that ecological restoration and WC measures have been highly effective in these areas, leading to significant protection of the region’s natural resources. Regarding Water Conservation Reserves (WCR), the study found that WCR have been increasing year by year, especially in the northern and northeastern regions. These areas are primarily flat plains, with extensive cultivated and developed land that is highly susceptible to human activities. Optimizing land use types and promoting the rational distribution of forests, grasslands, and construction land will further enhance WC in these areas.

Driving Factor Analysis: Through a combined analysis of the Deep Neural Network (DNN) model and the SHAP model, the study identified the key driving factors affecting WC. The results show that the DNN model can effectively predict the regional WC (R² > 0.90), with Land Use and Land Cover Change (LUCC) being the most influential factor. The increase in forest and grassland areas has a significant positive impact on WC. Precipitation and the drought index, within reasonable ranges, have the most significant positive effect on WC, while higher annual average temperatures and potential evapotranspiration suppress WC.

Spatiotemporal Distribution and Policy Implications: This study highlights the spatiotemporal changes in WC in Xiong’an New Area and emphasizes the critical role of ecological policies and management measures in enhancing WC. Notably, from 2015 to 2018, as ecological protection measures were advanced, the region’s WC capacity significantly improved, particularly in the areas of ecological restoration and land use optimization. The study underscores the importance of ecological restoration, vegetation recovery, and water resource protection policies in boosting WC. It identifies the northern and northeastern parts of Xiong’an New Area as having high ecological protection potential and recommends further optimizing the land use structure in these areas to promote sustainable water resource management.
